# Delayed effects of transcriptional responses in *Mycobacterium tuberculosis* exposed to nitric oxide suggest other mechanisms involved in survival

**DOI:** 10.1038/s41598-017-08306-1

**Published:** 2017-08-15

**Authors:** Teresa Cortes, Olga T. Schubert, Amir Banaei-Esfahani, Ben C. Collins, Ruedi Aebersold, Douglas B. Young

**Affiliations:** 1 Department of Pathogen Molecular Biology, Faculty of Infectious and Tropical Diseases, London School of Hygiene and Tropical Medicine, London, WC1E 7HT United Kingdom; 2 Mycobacterial Systems Biology Laboratory, The Francis Crick Institute, 1 Midland Road, London, NW1 1AT United Kingdom; 3 Department of Biology, Institute of Molecular Systems Biology, ETH Zurich, 8093 Zurich, Switzerland; 4 PhD Program in Systems Biology, Life Science Zurich Graduate School, University of Zurich and ETH Zurich, Zurich, Switzerland; 5 Faculty of Science, University of Zurich, 8057 Zurich, Switzerland; 6 MRC Centre for Molecular Bacteriology and Infection, Imperial College London, London, SW7 2AZ United Kingdom; 7 Department of Human Genetics, University of California, Los Angeles, Los Angeles, CA 90095 United States of America

## Abstract

*Mycobacterium tuberculosis* has succeeded as a human pathogen for tens of thousands of years thanks to its ability to resist and adapt to the adverse conditions it encounters upon infection. Bacterial adaptation to stress is commonly viewed in the context of transcriptional regulation, with the implicit expectation that an initial transcriptomic response is tightly coupled to an ensuing proteomic response. However, after challenging *M*. *tuberculosis* with nitric oxide we found that the rapid transcriptional responses, detectable within minutes of nitric oxide exposure, typically took several hours to manifest on the protein level. Furthermore, early proteomic responses were dominated by the degradation of a set of proteins, specifically those containing damaged iron-sulphur clusters. Overall, our findings are consistent with transcriptional responses participating mostly in late-stage recovery rather than in generating an immediate resistance to nitric oxide stress, suggesting that survival of *M*. *tuberculosis* under acute stress is contingent on mechanisms other than transcriptional regulation. These findings provide a revised molecular understanding of an important human pathogen.

## Introduction

During the course of infection, *Mycobacterium tuberculosis* passes through a series of intracellular and extracellular locations, from alveolar macrophages to granulomatous lesions, where it is exposed to various types of stress. Mechanisms of adaptation to such environmental stresses are an important focus in mycobacterial research^1^. The major experimental approach used to date has involved monitoring of transcriptional changes triggered by environmental perturbation or by gene disruption, alongside with systematic mapping of transcription factor binding sites^2–4^. This has led to a prevailing model in which adaptation is viewed as the consequence of a cascade of transcriptional signalling driving subsequent downstream changes to proteome and metabolome^2^. The aim of the present study was to test this model by the integrated, time resolved analysis of transcriptome and proteome responses of *M*. *tuberculosis* during exposure to the well-defined and clinically relevant environmental stress induced by nitric oxide (NO).

Nitric oxide is an agent which generates a systems-level stress response through its broad reactivity with multiple macromolecules, thereby providing an opportunity for detailed analysis of connectivity between transcription and translation^5^. Exposure to NO mimics the major microbicidal activity experienced by *M*. *tuberculosis* following uptake by mouse macrophages and represents a major component of the human immune response to *M*. *tuberculosis* ^6^. Intracellular release of NO is also an important anti-mycobacterial mechanism of a class of nitroimidazole drugs currently in late-stage development for treatment of tuberculosis^7^. Building on previously published models^8–10^, we set up an experimental system in which *M*. *tuberculosis* is exposed to NO at a concentration that causes transient arrest of cell growth over a 48-hour period. We compared the time resolved changes occurring at the level of the transcriptome and proteome and analysed the regulatory interactions to better understand *M*. *tuberculosis* survival to NO.

While we detected the anticipated programme of a transcription-driven response, translation of induced transcripts occurred over a time scale of several hours, with significant levels of new proteins becoming available only during the recovery phase of the stress response. We also observed direct and early changes to the proteome that were followed by compensatory transcriptional responses. Importantly, early changes in the proteome were driven by the degradation of damaged proteins containing iron-sulphur clusters, which are ubiquitous cofactors of enzymes composed of iron and inorganic sulphur. Overall, our results show that replacing the conventional transcription-driven model of mycobacterial adaptation by an integrated systems approach involving quantitative transcript and protein profiles, provides an improved understanding of the dynamic response of *M*. *tuberculosis* to acute stress.

## Results

### Responses to nitric oxide stress in *M*. *tuberculosis* occur on various molecular levels

**Figure 1:**
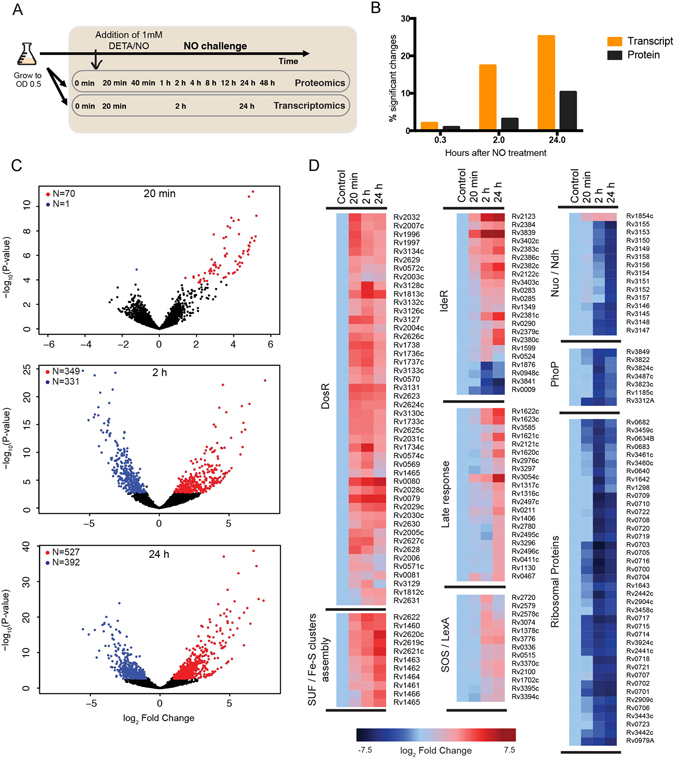
Impact of nitric oxide on the *Mycobacterium tuberculosis* transcriptome. (**A**) Diagram of the experimental procedure. Cultures were grown to mid-exponential phase and DETA/NO was added once at time point 0 to a final concentration of 1 mM. Samples for transcriptomic and proteomic analysis were harvested from the same cultures during a 48-hour time course at the indicated time points. All experiments were performed in triplicate. (**B**) Kinetics of transcript and protein changes in response to NO. Graph showing the percentage of significantly changing genes and proteins among the detectable transcriptome and proteome, respectively, in response to the challenge with NO. (**C**) Time course of transcriptional changes in response to NO. Volcano plots showing mRNA fold changes compared to time point 0 for all genes upon challenge with NO. Red corresponds to genes with >1 log_2_ fold differential expression and adjusted P-value < 0.01. Blue corresponds to genes with <−1 log_2_ fold differential expression and adjusted P-value < 0.01. (**D**) Transcriptional regulation in response to NO. Heat map of log_2_ fold changes for differentially expressed transcripts in response to NO grouped by relevant operons/regulons.

To study the time resolved transcriptional and translational *M*. *tuberculosis* response from initial survival to eventual escape from NO stress, we exposed exponentially growing *M*. *tuberculosis* cells to a sub-lethal concentration of NO and followed the adaptive response over 48 hours. Consistent with previously published data^8–10^, addition of 1 mM diethylenetriamine/nitric oxide (DETA/NO) transiently arrested growth of exponentially growing *M*. *tuberculosis*, but the cells retained viability and resumed growth upon addition of fresh medium after 48 hours of NO challenge (Supplementary Fig. S1). Samples were obtained from three replicate experiments and we sampled aliquots for transcriptome profiling by RNA sequencing^11^ at 20 min, 2 h and 24 h and for mass spectrometry-based shotgun proteomics^12^ at 20 min, 40 min, 1 h, 2 h, 4 h, 8 h, 12 h, 24 h and 48 h post NO exposure (Fig. 1A).

Exposure to NO in *M*. *tuberculosis* induced a stress response involving changes at transcript and protein level. Overall, we detected transcription of 3,286 genes (81.76% of the annotated genes) and identified significant changes in the expression of more than 1,200 of these genes, 705 being up-regulated and 559 down-regulated (adjusted P-value < 0.01, absolute log_2_ fold change >1) at one or more time points after challenge with NO compared to expression levels before exposure to NO (Fig. 1B, Supplementary Data S1). Furthermore, shotgun proteome analysis detected over 1,400 proteins (Supplementary Data S2) of which 123 were up-regulated and 140 down-regulated (adjusted P-value < 0.01, absolute log_2_ fold change >0.5) (Fig. 1B).

### Nitric oxide stress triggers immediate changes in gene expression

Changes in transcript patterns occurred rapidly after exposure to NO. Within 20 min of NO exposure, the expression of 70 genes had significantly increased by a factor of at least two-fold (adjusted P-value < 0.01; Fig. 1C, Supplementary Data S1), 37 of which were members of the well-characterised DosR/DevR regulon encoding a set of ~50 protein chaperones and enzymes that contribute to maintenance of cell viability during transition to a non-replicating state (Fig. 1D)^8, 13, 14^. Additionally, the SUF operon (Rv1460-Rv1466) encoding enzymes required for assembly of iron-sulphur clusters^15^ was up-regulated. While the activation of members of the DosR regulon was transient, with total reads mapping to DosR transcripts falling by almost 20% two hours after addition of NO, the SUF operon showed a more sustained transcriptional activation over the 24-hour time course (Fig. 1D, Supplementary Fig. S2).

NO can act as a potent reversible inhibitor of aerobic respiration in bacteria^8^. We found extensive evidence of restructuring of electron transport pathways in response to NO, both at the transcriptional and protein levels. For example, transcriptional activation of *ndh* (Rv1854c), encoding a non-proton pumping type II NADH dehydrogenase, was observed within 20 minutes of exposure to NO, though a significant rise in corresponding protein levels was detected only after 24 hours. Furthermore, there was evidence of transient transcriptional repression of the major succinate dehydrogenase operon (Rv0249c-Rv0247c)^16^, as well as up-regulation of *cydABCD* cytochrome bd oxidase operon (Rv1620c-Rv1623c). Overall, these findings suggest interference of NO with the aerobic respiratory chain.

Later transcriptional changes reflected the state of the metal household. For example, two hours after exposure to NO, there was evidence of iron deprivation, resulting in the up-regulation of 17 (out of 49) members of the IdeR regulon^17, 18^. In contrast, the opposite response was seen for copper, with up-regulation of genes – such as the *mymT* copper metallothionine^16^ – that are subject to repression by CsoR (Rv0967) and RicR (Rv0190) in the absence of free copper^19, 20^. De-repression of these genes could reflect an increase in free copper as a result of damage and repair of terminal oxidase heme-copper centres, or release of copper from some form of intracellular storage as recently described in *E*. *coli* ^21^. The absence of altered expression of the Zur regulon^22^ suggests that exposure to NO does not significantly disrupt zinc homeostasis.

Late transcriptional adaptation at the 24-hour time point featured differential expression of more than 900 genes where transcripts with increased abundance were linked to cellular recovery, including genes encoding DNA repair enzymes, such as the SOS-regulated DnaE2/ImuA’/ImuB low fidelity DNA polymerase^23^, and replacement of a subset of the proteins subject to NO-induced degradation as described below (Fig. 1D).

### Network analysis reveals key regulators of the transcriptional response to NO stress

**Figure 2:**
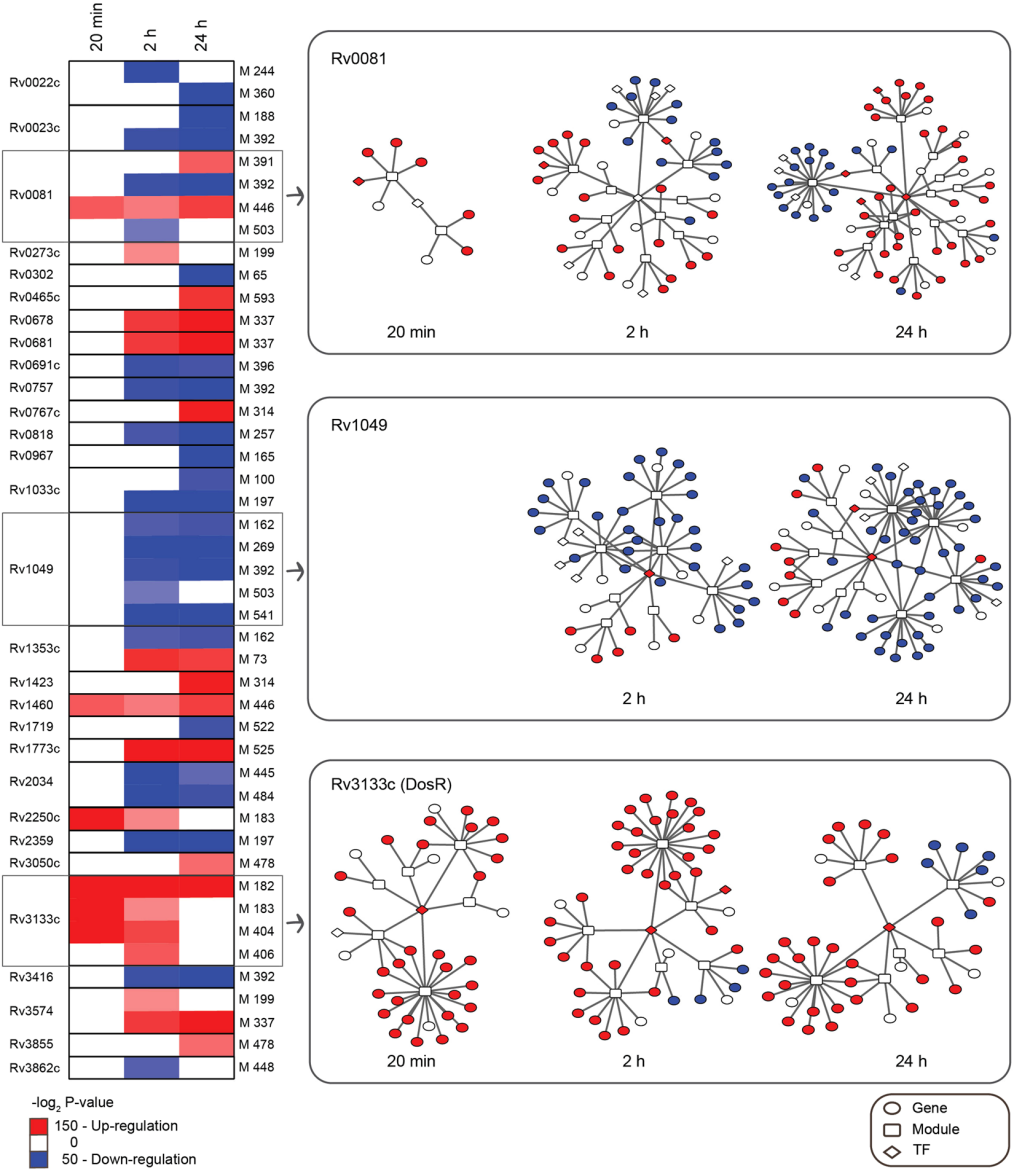
Module-centric view of the transcriptional regulatory network in response to nitric oxide stress. Heat map shows the adjusted and log_2_ transformed P-values for the association of a transcription factor with an EGRIN module. Red indicates that a given module is upregulated while blue indicates downregulated modules. White cells denote modules that did not meet the significance threshold to be considered enriched (adjusted P-value < 0.01). For each of the three main transcriptional regulators of the NO stress response, a summary of the sub-networks over time is illustrated. Details such as gene, module and transcription factor names are available in Supplementary Fig. S3.

To identify the transcription factors orchestrating the transcriptional responses of *M*. *tuberculosis* to the NO challenge in a more systematic way, we integrated our transcriptome-wide data with an existing regulatory network model. Specifically, we used the Environment and Gene Regulatory Influence Network (EGRIN) model^24^, which describes clusters of co-regulated transcripts (modules) and corresponding transcription factors^3, 4^. The differentially expressed genes from our transcriptomic data set were mapped on the EGRIN modules for each time point^25^ resulting in 13, 54 and 59 significantly enriched modules (adjusted P-value < 0.01) at 20 min, 2 h and 24 h post NO exposure, respectively (Supplementary Data S4). Next, the significantly enriched modules were linked to transcription factors, leading to the identification of 29 transcription factors associated with them in at least one time point (Fig. 2, Supplementary Data S4). The main regulators, as determined by the highest numbers of linking modules, were Rv0081, Rv1049 and DosR (Rv3133c). Concordant with the rapid transcriptomic response observed for members of the DosR regulon, the transcriptional sub-network of DosR responded first, whilst the sub-networks of Rv0081 and Rv1049 increased in complexity progressively over the time (Fig. 2; Supplementary Fig. S3). Regulators of the transcriptional response to NO include both transcriptional activators and repressors, with DosR dictating mainly upregulation and Rv1049 mainly downregulation of the genes in their respective sub-networks, whereas Rv0081 acted both by activating and repressing gene expression (Fig. 2; Supplementary Fig. S3). Overall, the regulatory network of *M*. *tuberculosis* in response to NO shared similar key regulators to the hypoxia response network previously characterised (e.g., DosR and Rv0081)^2^, however, we also identified regulators, such as the transcription factor Rv1049, that seem more specific to the NO response^26^.

### Protein level response is significantly delayed compared to transcript level response

A moderate correlation has been previously reported in exponentially growing cultures of *M*. *tuberculosis* between the levels of transcript and corresponding protein as measured by RNA sequencing reads and mass spectrometry ion counts^27^. In the current study, we found a similar correlation between mRNA and protein levels prior to the NO stress, with a spearman correlation coefficient (r) of 0.38. This correlation between mRNA and protein levels progressively decreased during the NO time course (Supplementary Fig. S4).

**Figure 3:**
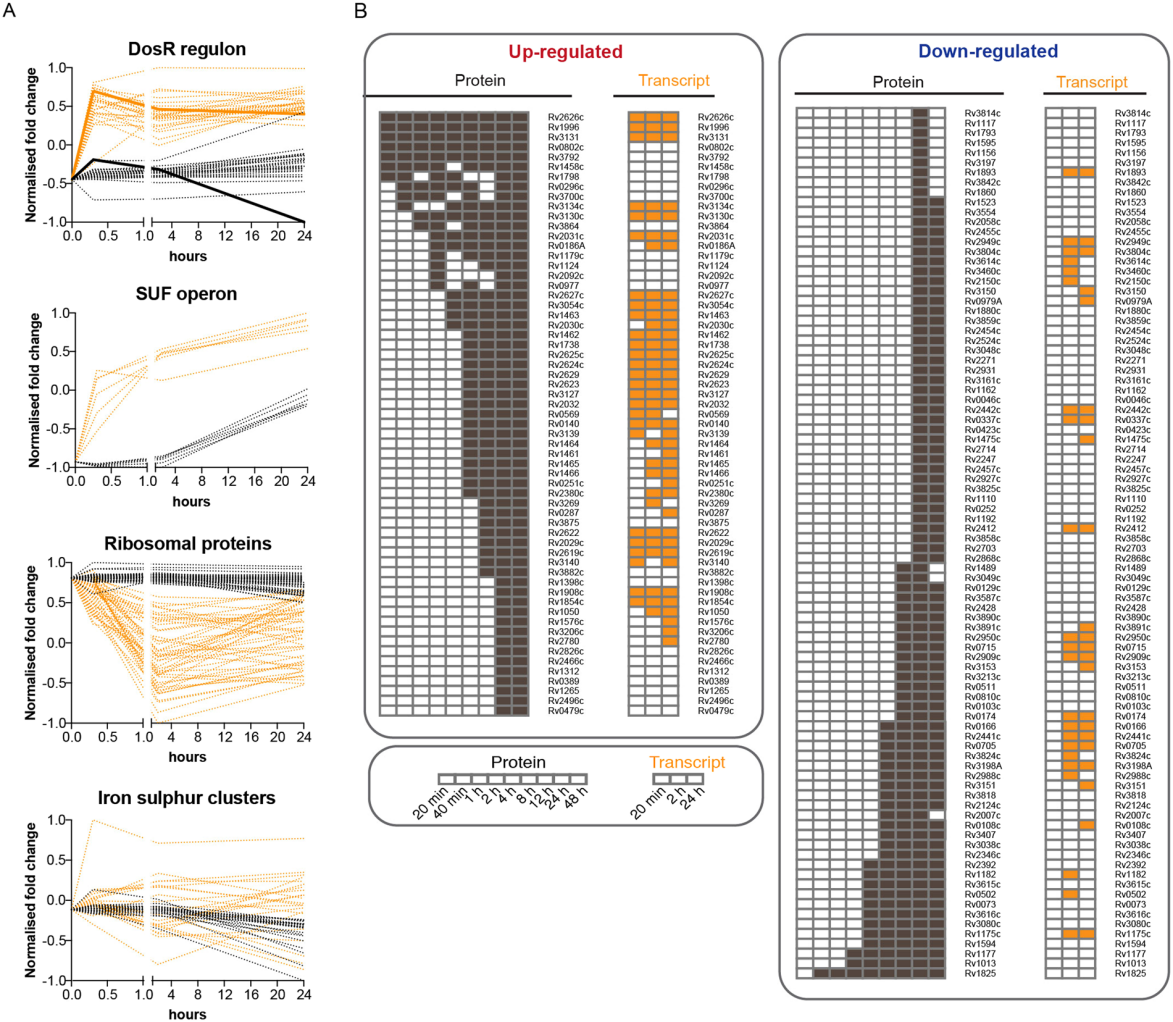
Role of transcriptional regulation for protein abundances during nitric oxide stress. (**A**) Line plots showing changes in transcript (orange) and protein (black) abundances over time in response to NO. The first panel shows the delayed induction of DosR proteins compared to the rapid transcriptional activation. The discordant response observed for FdxA (containing an iron-sulphur cluster) is highlighted with full lines. The second panel shows the delayed induction of proteins from the SUF operon. The third panel shows the delayed decrease in abundance of ribosomal proteins. The fourth panel shows the discordant response observed for proteins containing iron-sulphur clusters. In all panels, fold changes have been normalised so that the maximal and minimal fold change are 1 and minus 1, respectively. (**B**) Global dynamics of protein up-regulation and down-regulation in response to NO. Heat map shows the correlated dynamics of protein up-regulation and down-regulation with mRNA transcripts for the set of proteins with significant altered abundance 24 hours after exposure to NO. Black coloured cells indicate a significant change of protein abundance compared to time point 0 (log_2_ fold change >0.5; adjusted P-value < 0.01); white cells denote measurements that did not meet this threshold for differential expression. Right panels show the expression pattern at the mRNA transcript level. In this case, differentially expressed genes were called when log_2_ fold change >2 and adjusted P-value < 0.01 with significant changes indicated by orange coloured cells.

Although, mRNA transcript changes also partially correlated with protein changes during NO stress, the corresponding responses in the proteome occurred later with, for example, almost 20% of the transcriptome significantly changing after 2 hours compared to only three percent of the proteome (Fig. 1B). As illustrated in Fig. 3A, the immediate transcriptional response to NO stress with activation of the DosR regulon and the SUF operon is reflected on the level of proteins but delayed by several hours. In fact, while transcriptional activation of the early response genes peaked at 20 minutes, protein synthesis continued to increase over a 24-hour period. As discussed later, one important exception to this delayed protein synthesis was the iron-sulphur containing DosR-regulated protein FdxA (Rv2007c), that showed a sustained decrease in protein levels (represented as solid lines in the DosR Regulon panel in Fig. 3A). This delayed correlation between transcription and translation was also evident for the set of up-regulated proteins at 24 hours, with 40 (66%) of the up-regulated proteins associated with a statistically significant increase in the corresponding transcript levels but at earlier time points (Fig. 3B). Also, this delayed pattern between mRNA and protein levels was observable for down-regulated genes, for example in the case of ribosomal proteins (Fig. 3A, Ribosomal proteins panel). Overall, even though transcript levels were rapidly responding to NO stress, corresponding changes in protein levels manifested only hours after, suggesting that the rapid transcriptional activation is unlikely to play a primary role in the immediate response of *M*. *tuberculosis* to the NO challenge.

### Targeted degradation of iron-sulphur cluster proteins in response to NO

In general, the correlation between transcription and translation was lower in the case of down-regulated proteins at 24 hours compared to the set of up-regulated proteins previously mentioned (Fig. 3B). Down-regulation of 29 (33% of the down-regulated proteins at 24 hours) proteins, including ribosomal proteins, was preceded by a corresponding response in the transcriptome (Fig. 3A and B). Interestingly, the list of proteins with decreased abundance at 24 hours in the absence of a transcriptional change included 22 proteins predicted to contain iron-sulphur clusters, that are needed for proper enzymatic activity (Supplementary Data S3). As highlighted earlier, the discordant response between transcription and translation is particularly striking for the DosR-regulated ferredoxin FdxA (Rv2007c), containing one 4Fe-4S ferredoxin-type domain and whose transcript levels were strongly induced in response to NO but for which protein levels fell below the detection limit at 48 hours (Fig. 3A). Given the absence of change in transcript level, decreased abundance of iron-sulphur cluster proteins is likely to represent active degradation rather than decreased synthesis. Iron-sulphur clusters are known to be susceptible to attack by NO, generating potentially lethal long-lived dinitrosyl iron complexes (DNICs)^28^. Degradation of damaged iron-sulphur cluster proteins and export of DNICs represents a potential defence mechanism that is consistent with the observed proteome changes and with transcriptional evidence of iron deficiency and prolonged activation of the SUF operon required for regeneration of iron-sulphur clusters. We also looked at the effect of NO on other reported NO targets, such as hemoglobins^29^, cytochrome c oxidase^30^ or enzymes containing thiol groups^31–33^, however, we did not detect any evidence of a similarly striking degradation of these other classes of proteins, suggesting that the interaction of NO with these targets is reversible.

In summary, these findings support the notion that many cellular processes in response to NO stress are not accurately reflected by transcriptional changes alone and that it is important to include measurements at different molecular levels to obtain a complete and accurate picture of the bacterial response to stress.

## Discussion

In the present study, we found that exposure of *M*. *tuberculosis* to NO induces a systems-level stress response involving a complex time course of changes in RNA and protein levels. Transcriptional responses measured by RNA sequencing recapitulated the central features previously documented by microarray profiling^8–10^, with prominent up-regulation of the DosR regulon, disruption of iron metabolism, and reduced expression of ribosomal protein genes. By integrating data obtained from RNA sequencing with peptide mass spectrometry we were able to monitor the impact of transcriptional adaptation on the proteome. Comparison of up-regulated transcript-protein pairs demonstrated a strong qualitative correlation, but occurring over widely different time scales. While we observed rapid transcriptional up-regulation within 20 minutes of exposure to NO, translation into protein product occurred over a prolonged 24-hour time course. This is distinct from the conventional model of bacterial gene expression in which ribosomes attach to transcripts as they emerge from RNA polymerase, with transcription and translation following similar kinetics. A similar delay in protein production has also been described in bacteria^34–36^, yeast^37–39^ and mammalian systems^40^ outside steady-state conditions, highlighting the relevance that this mechanism may play during adaptation to dynamic processes. In the particular context of adaptation to NO in *M*.*tuberculosis*, a consequence of a slower translation is that the transcriptionally induced protein changes will be unable to influence the bacterium’s response to the primary challenge, but may contribute to late-stage recovery and to subsequent NO exposure resulting from sequential rounds of host cell lysis and re-phagocytosis. This would suggest that the functional biology determining survival or death depends on more rapid adjustments occurring below the radar of transcription-driven adaptation. Application of an analogous systems-based approach to study the response of *M*. *tuberculosis* to lower doses of NO, resembling the ones *M*. *tuberculosis* faces during infection, as well as other stress stimuli; will be important to address whether the delayed translation is a general phenomenon for slow-growing *M*. *tuberculosis* or a specific aspect of the response to NO.

A second novel finding is the observation of targeted protein degradation in response to NO. This had the most dramatic impact on proteins with iron-sulphur clusters and may reflect an important mechanism to avoid accumulation of potentially lethal dinitrosyl iron complexes resulting from NO attack. A requirement to clear damaged proteins may explain the enhanced sensitivity to NO observed for proteasome mutants of *M*. *tuberculosis* ^41^. One consequence of this phenomenon is an inverse correlation between mRNA and protein for a subset of genes; an example is the increased abundance of *fdxA* transcripts but decreased abundance of FdxA protein. NO is also known to nitrosylate protein thiol groups^31^. However, we did not detect decreased abundances of proteins belonging to the S-nitroso proteome suggesting that, in contrast to iron-sulphur cluster proteins, repair of nitrosylated thiol groups does not involve protein degradation.

Overall, our data suggest that an adaptive response driven by transcriptional regulation is unlikely to determine the immediate survival of *M*. *tuberculosis* exposed to acute stress. Post-translational modification, including protein degradation, and allosteric regulation of enzyme function provide the potential for more rapid responses that are likely to be at least as important as transcriptional regulation. Therefore, integrated analyses including different classes of biomolecules are required to obtain a better understanding of the biology of infection and enhance the discovery of drugs with novel mechanisms of action.

## Methods

### Culture conditions and sample generation

*Mycobacterium tuberculosis* H37Rv (SysteMTb) was grown in Middlebrook 7H9 medium supplemented with 0.4% glycerol, 0.085% NaCl, 0.5% BSA and 0.05% Tyloxapol in roller bottle culture (2 rpm at 37 °C). For nitric oxide experiments, cultures were grown until mid-exponential phase and Diethylenetriamine/nitric oxide adduct (DETA/NO, Sigma) was added to a final concentration of 1 mM. Samples for transcriptomic and proteomic analyses were harvested from the same cultures. Experiments were performed in triplicates. All work involving live *M*. *tuberculosis* was performed in a dedicated Biosafety Level 3 (BSL 3) laboratory.

### Transcriptomics

For transcriptomic analyses, samples were harvested immediately before addition of nitric oxide and 20 minutes, 2 and 24 hours after nitric oxide addition. For each sample, 25 mL of culture were spun down and isolation of RNA was performed using the FastRNA Pro blue kit from QBiogene/MP Bio according to manufacturer’s instructions. All RNA samples were treated with Turbo DNase free (Ambion) until any DNA contamination removed. Concentration and quality control of RNA samples was measured by Nanodrop (ND-1000, Labtech) and Agilent RNA chip (2100 Bioanalyser).

For construction of directional RNA-seq libraries, Ribo-Zero™ and ScriptSeq™ v2 Kits (Epicentre) were used. Briefly, 5 µg of total RNA were used for ribosomal RNA removal using the Ribo-Zero rRNA removal kit (Gram-Positive Bacteria) following manufacturer’s instructions. 50 ng of Ribo-Zero-treated RNA were used to construct cDNA libraries for RNA sequencing using the ScriptSeq™ v2 Kit. RNA-seq libraries were sequenced as single-end reads on an Illumina HiSeq 2000 sequencer.

Raw reads were first filtered to discard low quality reads. FastQC (Babraham Bioinformatics) was used to inspect read base quality scores. Poor quality read bases were trimmed using the SolexaQA package^42^; default parameters were used, trimming bases with confidences p > 0.05, and removing reads <25 bases. Reference based mapping using the reference genome of *M*. *tuberculosis* H37Rv [EMBL:AL123456] was performed with BWA^43^. Genome coverage, defined as number of reads mapped per base of H37Rv genome, was calculated using BEDTools^44^. Normalised reads for each library were computed using DESeq2^45^. For the identification of background levels of expression within each library, the distribution of log_2_ normalised reads across the libraries was plotted. Plotting of log_2_ normalised reads followed a Poisson distribution and the 10^th^ percentile of normalised reads was used as a cut-off for gene expression.

For differential expression analyses, genome coverage of reads mapping to genes were used for statistical testing using DESeq2^45^, a method based on the negative binomial distribution and implemented in the R statistical environment. Differentially expressed genes were considered when fold changes were greater or equal than two-fold and the corresponding adjusted P-value was less than 0.01. To model the transcriptional data, we used the EGRIN model^24^. We tested differentially expressed genes (fold change >2, P-value < 0.01) for enrichment in the EGRIN modules with the hypergeometric test. Significantly enriched modules (Benjamini-Hochberg adjusted P-value < 0.01) and the predicted regulating TFs based on the ChIPSeq and TFOE data were reported as the results of the model^3, 4^. Thereafter, we estimated false discovery rate of the identified TFs using a scrambled transcriptional dataset. To generate the scrambled dataset, the gene names of the transcriptomic dataset were shuffled and none of the EGRIN modules were enriched significantly.

### Proteomics

For proteomic analyses, samples were harvested from the same triplicate cultures immediately before addition of NO and 20, 40 minutes, 1, 2, 4, ﻿8, 12, 24 and 48 hours after NO addition. For each sample, 25 mL of culture were spun down. Bacterial cell pellets were dissolved in lysis buffer containing 8 M Urea and 0.1% RapiGest (Waters) in 0.1 M ammonium bicarbonate buffer and were disrupted by applying two 40-second cycles with FastPrep®-24 (MP Biomedicals). Protein concentration was determined using a BCA assay according to manufacturer’s protocol (Thermo Fisher Scientific). Protein disulfide bonds were reduced by tris(2-carboxyethyl)phosphine (TCEP) and the resulting free cysteine residues were alkylated by iodoacetamide. Excessive iodoacteamide was captured by addition of N-acetyl cysteine. Extracted protein samples were diluted with ammonium bicarbonate buffer to reach a urea concentration of <2 M and then digested with sequencing-grade modified trypsin (Promega). To stop the tryptic digest and to precipitate RapiGest the pH was lowered to 2 using 50% trifluoro acetic acid (TFA). Water-immiscible degradation products of RapiGest were pelleted by centrifugation and the cleared peptide solution was desalted with C18 reversed-phase columns (Sep-Pak Vac C18, Waters), dried under vacuum, and re-solubilised to a final concentration of 1 mg/ml.

One µg of each peptide sample was analysed on a nano-LC system (Eksigent Technologies) connected to an LTQ Orbitrap XL mass spectrometer equipped with a nanoelectrospray ion source (Thermo Fisher Scientific). Peptides were separated on a fused silica microcapillary column (10 cm × 75 µm, New Objective) packed in-house with C18 resin (Magic C18 AQ 3 µm diameter, 200 Å pore size, Michrom BioResources) with a linear gradient from 95% solvent A (2% acetonitrile/0.1% formic acid) and 2% solvent B (98% acetonitrile/0.1% formic) to 35% solvent B over 90 min at a flow rate of 300 nl/min. The data acquisition mode was set to obtain one MS1 scan in the orbitrap at a resolution of 60,000 full width at half maximum followed by collision induced dissociation of the five most abundant precursor ions with a dynamic exclusion for 30 s. MS2 spectra were acquired in the linear ion trap.

Thermo raw files were converted into mzXML format using ProteoWizard^46^. The acquired MS2 spectra were searched with OMSSA^47^ and XTandem^48^ against an *M*. *tuberculosis* H37Rv protein database (TubercuList v2.6) additionally containing reversed sequences of all proteins in the database. Search parameters were as follows: semi-tryptic peptides (proteolytic cleavage after lysine and arginine unless followed by proline) and maximally one missed cleavage were allowed, mass tolerance for the precursor ions was set to 15 ppm and for the fragment ion to 0.4 Da. Carbamidomethylation at cysteines was set as a fixed modification. The output of the search engine was processed using PeptideProphet and iProphet as part of the TPP^49, 50^. Only peptides at a false discovery rate of less than 1% were taken into consideration for further analysis. For MS1-based label-free quantification the openMS framework was used^51^. Signals were normalised on peptide feature level such that the median signal in each sample is the same, assuming that the amount of total protein per cell does not change significantly during the experiment. Abundances of the three most intense peptides per protein were averaged to get a protein abundance value. The same peptides were used for protein quantification across all samples and proteins with less than three peptides were included; in cases where only a single peptide per protein was detectable, the value of that peptide was taken as a protein value. For relative quantification (i.e. fold changes compared to time point 0), the R package MSstats^52^ was used. All peptide precursors with missing values in more than 9 out of the 30 samples were removed before analysis. MSstats was run using fixed effects models to determine fold changes and P-values. P-values were corrected for multiple testing with the Benjamini-Hochberg method.

### Data availability

The transcriptomic data that support the findings of this study is available in ArrayExpress (www.ebi.ac.uk/arrayexpress) under accession number E-MTAB-5557. The mass spectrometry proteomics data have been deposited to the ProteomeXchange Consortium via the PRIDE^53^ partner repository with the dataset identifier PXD006039.

## Electronic supplementary material

Supplementary Information

Dataset S1

Dataset S2

Dataset S3

Dataset S4

## References

[1] Barry CE (2009). The spectrum of latent tuberculosis: rethinking the biology and intervention strategies. Nat. Rev. Microbiol..

[2] Galagan JE (2013). The *Mycobacterium tuberculosis* regulatory network and hypoxia. Nature.

[3] Rustad TR (2014). Mapping and manipulating the *Mycobacterium tuberculosis* transcriptome using a transcription factor overexpression-derived regulatory network. Genome Biol..

[4] Minch KJ (2015). The DNA-binding network of *Mycobacterium tuberculosis*. Nat. Commun..

[5] Robinson JL, Adolfsen KJ, Brynildsen MP (2014). Deciphering nitric oxide stress in bacteria with quantitative modeling. Curr. Opin. Microbiol..

[6] Nathan C (2006). Role of iNOS in human host defense. Science (New York, N.Y.).

[7] Singh R (2008). PA-824 kills nonreplicating *Mycobacterium tuberculosis* by intracellular NO release. Science.

[8] Voskuil MI (2003). Inhibition of respiration by nitric oxide induces a *Mycobacterium tuberculosis* dormancy program. J. Exp. Med..

[9] Voskuil MI, Bartek IL, Visconti K, Schoolnik GK (2011). The response of *Mycobacterium tuberculosis* to reactive oxygen and nitrogen species. Front. Microbiol..

[10] Ohno H (2003). The effects of reactive nitrogen intermediates on gene expression in *Mycobacterium tuberculosis*. Cell. Microbiol..

[11] Arnvig, K. B. *et al*. Sequence-based analysis uncovers an abundance of non-coding RNA in the total transcriptome of *Mycobacterium tuberculosis*. *PLoS Pathog*. **7** (2011). 10.1371/journal.ppat.1002342 PMC3207917 22072964

[12] Aebersold R, Mann M (2016). Mass-spectrometric exploration of proteome structure and function. Nature.

[13] Mehra, S. *et al*. The DosR Regulon Modulates Adaptive Immunity and is Essential for *Mycobacterium tuberculosis* Persistence. *Am*. *J*. *Respir*. *Crit*. *Care Med*., doi:10.1164/rccm.201408-1502OC (2015). 10.1164/rccm.201408-1502OC PMC4451619 25730547

[14] de Majumdar S (2012). Appropriate DevR (DosR)-mediated signaling determines transcriptional response, hypoxic viability and virulence of *Mycobacterium tuberculosis*. PLoS One.

[15] Huet G, Daffé M, Saves I (2005). Identification of the *Mycobacterium tuberculosis* SUF machinery as the exclusive mycobacterial system of [Fe-S] cluster assembly: Evidence for its implication in the pathogen’s survival. J. Bacteriol..

[16] Cook, G. M., Hards, K., Vilchèze, C., Hartman, T. & Berney, M. Energetics of Respiration and Oxidative Phosphorylation in Mycobacteria. *Microbiol*. *Spectr*. **2**, doi:10.1128/microbiolspec.MGM2-0015-2013 (2014). 10.1128/microbiolspec.MGM2-0015-2013 PMC4205543 25346874

[17] Gold B, Marcela Rodriguez G, Marras SAE, Pentecost M, Smith I (2001). The *Mycobacterium tuberculosis* ideR is a dual functional regulator that controls transcription of genes involved in iron acquisition, iron storage and survival in macrophages. Mol. Microbiol..

[18] Pandey R, Rodriguez GM (2014). IdeR is required for iron homeostasis and virulence in *Mycobacterium tuberculosis*. Mol. Microbiol..

[19] Festa RA (2011). A novel copper-responsive regulon in *Mycobacterium tuberculosis*. Mol. Microbiol..

[20] Liu T (2007). CsoR is a novel *Mycobacterium tuberculosis* copper-sensing transcriptional regulator. Nat. Chem. Biol..

[21] Fung DKC, Lau WY, Chan WT, Yan A (2013). Copper efflux is induced during anaerobic amino acid limitation in *Escherichia coli* to protect iron-sulfur cluster enzymes and biogenesis. J. Bacteriol..

[22] Macia̧g A (2007). Global analysis of the *Mycobacterium tuberculosis* Zur (FurB) regulon. J. Bacteriol..

[23] Warner DF (2010). Essential roles for imuA’- and imuB-encoded accessory factors in DnaE2-dependent mutagenesis in *Mycobacterium tuberculosis*. Proc. Natl. Acad. Sci. USA.

[24] Peterson EJR (2014). A high-resolution network model for global gene regulation in *Mycobacterium tuberculosi*s. Nucleic Acids Res..

[25] Peterson EJR, Ma S, Sherman DR, Baliga NS (2016). Network analysis identifies Rv0324 and Rv0880 as regulators of bedaquiline tolerance in *Mycobacterium tuberculosis*. Nat. Microbiol..

[26] Brugarolas P (2012). The oxidation-sensing regulator (MosR) is a new redoxdependent transcription factor in *Mycobacterium tuberculosis*. J. Biol. Chem..

[27] Cortes T (2013). Genome-wide Mapping of Transcriptional Start Sites Defines an Extensive Leaderless Transcriptome in *Mycobacterium tuberculosis*. Cell Rep..

[28] Hickok JR (2011). Dinitrosyliron complexes are the most abundant nitric oxide-derived cellular adduct: Biological parameters of assembly and disappearance. Free Radic. Biol. Med..

[29] Davidge, K. S. & Dikshit, K. L. Haemoglobins of Mycobacteria: Structural features and biological functions. *Advances in Microbial Physiology* **63**, (Copyright © 2013 Elsevier Ltd. All rights reserved., 2013). 10.1016/B978-0-12-407693-8.00005-4 24054797

[30] Sarti P, Forte E, Mastronicola D, Giuffrè A, Arese M (2012). Cytochrome c oxidase and nitric oxide in action: Molecular mechanisms and pathophysiological implications. Biochim. Biophys. Acta - Bioenerg..

[31] Rhee KY, Erdjument-Bromage H, Tempst P, Nathan CF (2005). S-nitroso proteome of *Mycobacterium tuberculosis*: Enzymes of intermediary metabolism and antioxidant defense. Proc. Natl. Acad. Sci. USA.

[32] Lu J, Holmgren A (2014). The thioredoxin antioxidant system. Free Radic. Biol. Med..

[33] Newton GL, Buchmeier N, Fahey RC (2008). Biosynthesis and functions of mycothiol, the unique protective thiol of Actinobacteria. Microbiol. Mol. Biol. Rev..

[34] Jayapal, K. P. *et al*. Uncovering genes with divergent mRNA-protein dynamics in *Streptomyces coelicolor*. *PLoS One* **3** (2008). 10.1371/journal.pone.0002097 PMC2367054 18461186

[35] Hahne H (2010). A comprehensive proteomics and transcriptomics analysis of *Bacillus subtilis* salt stress adaptation. J. Bacteriol..

[36] Maier T (2014). Quantification of mRNA and protein and integration with protein turnover in a bacterium. Mol. Syst. Biol..

[37] Vogel C, Silva GM, Marcotte EM (2011). Protein expression regulation under oxidative stress. Mol. Cell. Proteomics.

[38] Lee MV (2014). A dynamic model of proteome changes reveals new roles for transcript alteration in yeast. Mol. Syst. Biol..

[39] Fournier ML (2010). Delayed Correlation of mRNA and Protein Expression in Rapamycin-treated Cells and a Role for Ggc1 in Cellular Sensitivity to Rapamycin. Mol. Cell. Proteomics.

[40] Jovanovic M (2015). Dynamic profiling of the protein life cycle in response to pathogens. Science.

[41] Darwin KH, Ehrt S, Gutierrez-Ramos J-C, Weich N, Nathan CF (2003). The proteasome of *Mycobacterium tuberculosis* is required for resistance to nitric oxide. Science.

[42] Cox MP, Peterson DA, Biggs PJ (2010). SolexaQA: At-a-glance quality assessment of Illumina second-generation sequencing data. BMC Bioinformatics.

[43] Li H, Durbin R (2009). Fast and accurate short read alignment with Burrows-Wheeler transform. Bioinformatics.

[44] Quinlan AR, Hall IM (2010). BEDTools: A flexible suite of utilities for comparing genomic features. Bioinformatics.

[45] Love MI, Huber W, Anders S (2014). Moderated estimation of fold change and dispersion for RNA-seq data with DESeq 2. Genome Biol..

[46] Kessner D, Chambers M, Burke R, Agus D, Mallick P (2008). ProteoWizard: open source software for rapid proteomics tools development. Bioinformatics.

[47] Barsnes H, Huber S, Sickmann A, Eidhammer I, Martens L (2009). OMSSA Parser: An open-source library to parse and extract data from OMSSA MS/MS search results. Proteomics.

[48] Teleman J (2014). Numerical compression schemes for proteomics mass spectrometry data. Mol. Cell. Proteomics.

[49] Keller A, Nesvizhskii AI, Kolker E, Aebersold R (2002). Empirical statistical model to estimate the accuracy of peptide identifications made by MS/MS and database search. Anal. Chem..

[50] Shteynberg D (2011). iProphet: Multi-level Integrative Analysis of Shotgun Proteomic Data Improves Peptide and Protein Identification Rates and Error Estimates. Mol. Cell. Proteomics.

[51] Weisser H (2013). An automated pipeline for high-throughput label-free quantitative proteomics. J. Proteome Res..

[52] Choi M (2014). MSstats: an R package for statistical analysis of quantitative mass spectrometry-based proteomic experiments. Bioinformatics.

[53] Vizcaino JA (2016). Update of the PRIDE database and its related tools. Nucleic Acids Res..

