# Supplementary material for: Delayed effects of transcriptional responses in Mycobacterium tuberculosis exposed to nitric oxide suggest other mechanisms involved in survival
Source: Sci Rep. 2017 Aug 15;7:8208. doi: 10.1038/s41598-017-08306-1 (PMC5557973; doi:10.1038/s41598-017-08306-1)
Supplement: Supplementary file 1 — Supplementary Information [file 41598_2017_8306_MOESM1_ESM.pdf]

Supplementary information

Delayed effects of transcriptional responses in *Mycobacterium tuberculosis* exposed to nitric oxide suggest other mechanisms involved in survival

Teresa Cortes[1,2,*], Olga T. Schubert[3,†], Amir Banaei-Esfahani[3,4], Ben C. Collins[3], Ruedi Aebersold[3,5] and Douglas B. Young[2,6].

[1]Department of Pathogen Molecular Biology, Faculty of Infectious and Tropical Diseases, London School of Hygiene and Tropical Medicine, London, WC1E 7HT, United Kingdom

[2]Mycobacterial Systems Biology Laboratory, The Francis Crick Institute, 1 Midland Road, London, NW1 1AT, United Kingdom

[3]Department of Biology, Institute of Molecular Systems Biology, ETH Zurich, 8093 Zurich, Switzerland

[4]PhD Program in Systems Biology, Life Science Zurich Graduate School, University of Zurich and ETH Zurich, Zurich, Switzerland

[5]Faculty of Science, University of Zurich, 8057 Zurich, Switzerland

[6]MRC Centre for Molecular Bacteriology and Infection, Imperial College London, SW7 2AZ, United Kingdom

[†]Present address: Department of Human Genetics, University of California Los Angeles, Los Angeles, CA 90095, United States of America

[*]Corresponding author: teresa.cortes@lshtm.ac.uk

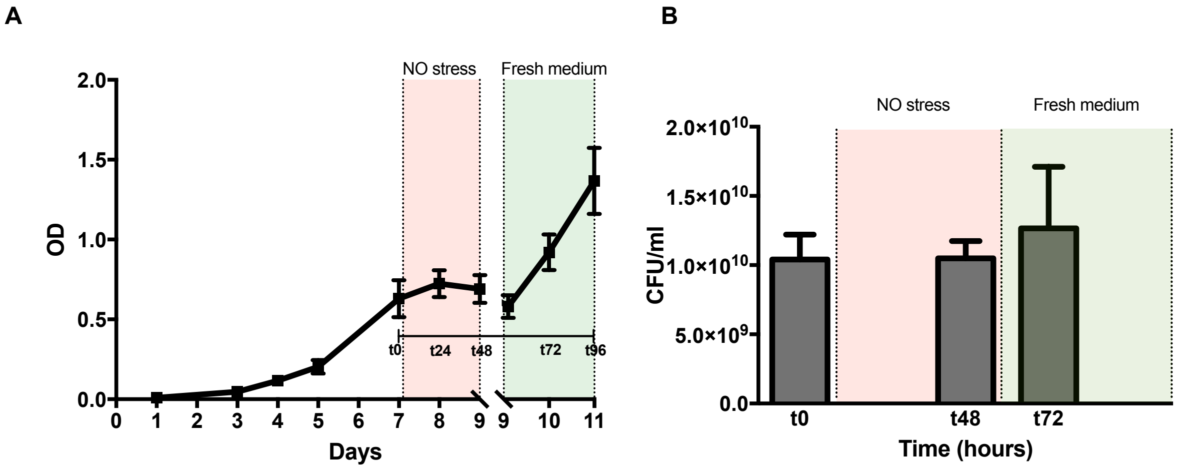

Figure S1. Effect of DETA/NO on growth of *M. tuberculosis*. Growth of *M. tuberculosis* during NO treatment was measured by optical density (OD, **A**) and colony forming units (CFUs, **B**). Addition of NO caused a transient growth arrest during the 48 hours time-course (demarcated by a red box) but cells retained viability and growth resumed on addition of fresh medium (demarcated by a green box). Corresponding CFU/ml were only calculated after 48 hours of NO stress and 24 hours after resuspension in fresh medium (referred as t72). Each point corresponds to the average of 3 biological replicates ± standard deviation.

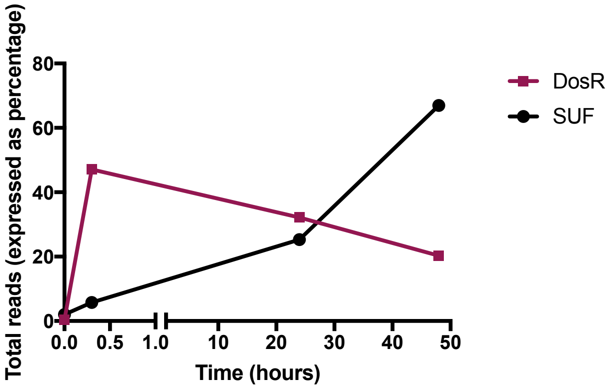

Figure S2. Kinetics of transcript accumulation for the DosR regulon and SUF operon genes after exposure to NO. Line plots showing accumulation of reads (expressed as percentage) for genes belonging to the DosR regulon (red) and SUF operon (black) during challenge with NO.

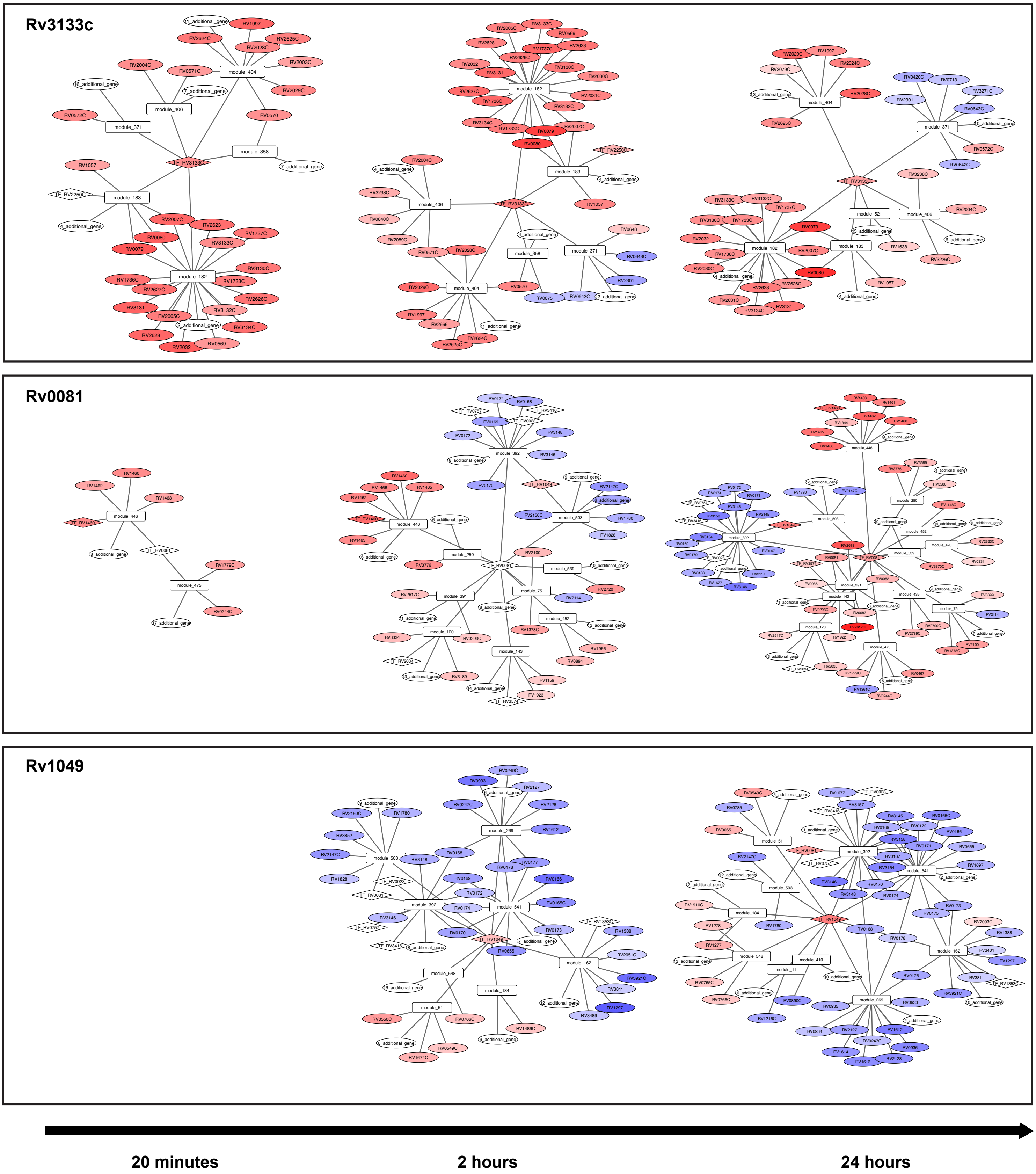

Figure S3. The sub-networks of DosR (Rv3133c), Rv0081 and Rv1049 in response to NO. Genes are represented by ovals, transcription factors by diamonds and modules by rectangles. Edges are coloured by P-value with red representing up-regulation and blue representing down-regulation.

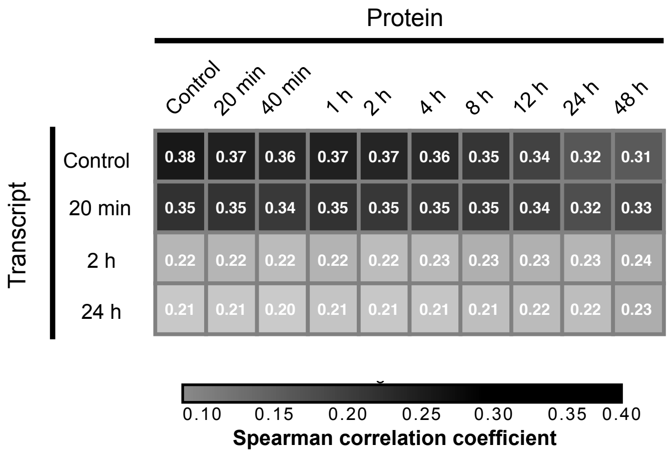

Figure S4. Pairwise Spearman correlation coefficients between transcript expression and protein abundance in response to NO. The strongest correlation between transcript and protein abundances was found in control samples, i.e. prior to the stress with NO.
